# Antibacterial activities of coumarin-3-carboxylic acid against *Acidovorax citrulli*

**DOI:** 10.3389/fmicb.2023.1207125

**Published:** 2023-09-20

**Authors:** Fa-Di Zhu, Xin Fu, Huo-Chun Ye, Hai-Xin Ding, Liu-Shuang Gu, Jing Zhang, Yong-Xia Guo, Gang Feng

**Affiliations:** ^1^Environment and Plant Protection Institute, Chinese Academy of Tropical Agricultural Science, Haikou, China; ^2^College of Agronomy, Heilongjiang Bayi Agricultural University, Daqing, China; ^3^Key Laboratory of Monitoring and Control of Tropical Agricultural and Forest Invasive Alien Pests, Ministry of Agriculture, Haikou, China; ^4^Key Laboratory of Organic Chemistry, Institute of Organic Chemistry, Jiangxi Science and Technology Normal University, Nanchang, China; ^5^Key Laboratory of Low-Carbon Green Agriculture in Northeastern China of Ministry of Agriculture and Rural Affairs, Daqing, China

**Keywords:** coumarin-3-carboxylic acid, bactericidal activity, mechanism, biofilm, *Acidovorax citrulli*

## Abstract

Coumarin-3-carboxylic acid (3-CCA), previously screened from natural coumarins, was found to possess strong antibacterial activity against *Acidovorax citrulli* (*Ac*). In order to further evaluate the activity of this compound against plant bacterial pathogens and explore its potential value as a bactericidal lead compound, the activity of 3-CCA against 14 plant pathogenic bacteria *in vitro* and *in vivo* was tested. Results showed that 3-CCA exhibited strong *in vitro* activities against *Ac, Ralstonia solanacearum, Xanthomonas axonopodis* pv. *manihotis, X. oryzae* pv. *oryzae*, and *Dickeya zeae* with EC_50_ values ranging from 26.64 μg/mL to 40.73 μg/mL. *Pot experiment results* showed that 3-CCA had powerful protective and curative effects against *Ac*. In addition, the protective efficiency of 3-CCA was almost equivalent to that of thiodiazole copper at the same concentration. The results of SEM and TEM observation and conductivity tests showed that 3-CCA disrupted the integrity of the cell membrane and inhibited polar flagella growth. Furthermore, 3-CCA resulted in reductions in motility and extracellular exopolysaccharide (EPS) production of *Ac* while inhibiting the biofilm formation of *Ac*. These findings indicate that 3-CCA could be a promising natural lead compound against plant bacterial pathogens to explore novel antibacterial agents.

## 1. Introduction

The bacterial diseases of crop plants have been major threats to crop growth, which can result in enormous losses in global food production and cause food security problems (Savary et al., 2012; Sundin et al., 2016). For example, bacterial fruit blotch caused by *Acidovorax citrulli* (*Ac*) has severely cut production in Cucumis (e.g., as high as 90% of yield loss in several watermelon cultivars) (Bahar and Burdman, 2010; Tian et al., 2015; Preston et al., 2021). Moreover, bacterial diseases caused by *Ralstonia solanacearum* (*Rs*), *Xanthomonas axonopodis* pv. *citri* (*Xac*), and *Xanthomonas oryzae* pv. *oryzae* (*Xoo*) are also recognized as destructive diseases that threaten crop production (Tondo et al., 2013; Jia et al., 2020; Qutb et al., 2020). At present, the control of plant bacterial diseases mainly depends on the use of copper pesticides and antibiotics, resulting in environmental pollution and antibacterial resistance (Vanneste et al., 2013; Sundin et al., 2016; Long et al., 2019). With attention on the threat to human health due to the irrational use of antibiotics, streptomycin was been officially withdrawn from the Chinese market in 2016 (Shang et al., 2020). Therefore, developing environment-friendly pesticides for controlling plant bacterial disease is a major challenge for chemists and biologists.

Coumarins are important natural compounds that are isolated from plants in the families of *Rutaceae, Asteraceae, Umbelliferae*, and *Leguminosae* sp. (Curini et al., 2006; Zubair et al., 2017), which exhibit multiple biological functions, including anticancer (Thomas et al., 2017), antituberculosis (Keri et al., 2015), antioxidation (Al-Majedy et al., 2016), anti-HIV (Yu et al., 2003), anti-inflammatory (Hadjipavlou-Litinaa et al., 2007), and antibacterial (Chen et al., 2016). In recent years, many coumarin derivatives have been reported to possess antibacterial activity. For instance, the hydroxycoumarins (esculetin and daphnetin) displayed superior activity against *Rs* by inhibiting the expression of lipopolysaccharide biosynthesis genes (Yang et al., 2016, 2021a). Notably, 6-methylcoumarin showed strong *in vitro* antibacterial activity against *Rs*, which involved cell elongation, disruption of cell division, and inhibition of the expression of genes (*ftsZ*) encoding bacterial division protein (Yang et al., 2021b). Therefore, coumarin was perceived as a privileged structure for designing new bactericides.

Coumarin-3-carboxylic acid (3-CCA) is an important derivative of coumarin, which has received great attention due to its diverse biological functions, including antitumor (Zhang et al., 2018), anticancer (Chimenti et al., 2004; Wei et al., 2017; Ji et al., 2021), and other antimicrobial properties (Lin et al., 2012; Liu et al., 2018). Our preliminary screening experiments confirmed that 3-CCA had significant inhibitory activity against some plant bacteria (data not shown). Therefore, we further conducted a bactericidal bioassay of 3-CCA against 14 phytopathogenic bacteria to explore its bactericidal spectrum. Specifically, assessments included *in vitro* and *in vivo* antibacterial activity, ultrastructure observation, and changes in pathogenicity-related physiological and biochemical indices.

## 2. Materials and methods

### 2.1. Chemicals

Coumarin-3-carboxylic acid (a.i. 97%, SKU No.: C856312-5g, Lot#:C12735407) was purchased from Shanghai Macklin Biochemical Technology Co., Ltd. (Shanghai, China). Commercial thiodiazole copper (a.i. 20% SC) was purchased from Zhejiang Longwan Chemicals Co., Ltd. (Wenzhou, China).

### 2.2. Pathogens

Fourteen phytopathogenic bacterial strains assayed for *in vitro* antibacterial screening were *Ac* (bacterial fruit blotch), *Xoo* (bacterial leaf blight of rice), *X*. *oryzae* pv. *oryzicola* (Xoc, bacterial leaf streak of rice), *Xac* (citrus bacterial canker), *X*. *campestris* pv. *mangiferaeindicae* (*Xcm*, bacterial black spot of mango), *Pseudomonas syringae* pv. *lachrymans* (*Psl*, cucurbit angular leaf spot), *Rs* (bacterial wilt of tomato), *P*. *syringae* pv. *actinidiae* (*Psa*, bacterial canker of kiwifruit), *Pectobacterium carotovorum* subsp. *brasiliense* (*Pcb*, bacterial soft rot of potato), *P*. *carotovorum* subsp. *carotovorum* (*Pcc*, bacterial soft rot of Chinese cabbage), *X*. *axonopodis* pv. *manihotis* (*Xam*, bacterial blight of cassava), *Dickeya zeae* (*Dz*, bacterial soft rot of banana), *X*. *fragariae* (*Xf* , bacterial angular leaf spot of strawberry), and *X*. *campestris* pv. *campestris* (*Xcc*, black rot of cabbage). All the strains were kindly provided by different researchers and then long-term preserved in our lab in 20% glycerol at −80°C for further use. Strains were cultured on Luria–Bertani agar (LA) plates (containing 10 g of tryptone, 5 g of yeast extract, 10 g of NaCl, 16 g of agar, and 1 L of distilled water) or in LB broth (without agar) at 28°C in the dark.

### 2.3. *In vitro* antibacterial assay

The abovementioned 14 plant pathogenic bacterial strains were used for the antibacterial bioassay according to the method described by Li et al. (2014). All strains were first transferred and cultured on LA plates, then single bacterial colonies were inoculated in LB broth at 28°C and 180 rpm until OD_600_ reached 0.6. The experiments were conducted on 96-well plates. Ten microliters of bacterial cultures were inoculated in 190 μL of LB broth in each well that contained 3-CCA with final concentrations of 100, 50, or 25 μg/mL. Acetone (1%) was used as a solvent control. All 96-well plates were incubated at 28°C and 180 rpm for 12 h. Then, the bacterial growth was investigated by measuring the OD_600_ with a microplate reader (Synergy H1, BioTek Instruments Inc., Vermont, USA).

The toxicities of 3-CCA to five plant pathogenic bacteria, including *Ac, Dz, Rs, Xam*, and *Xoo*, were tested at concentrations of 100, 50, 25, 12.5, and 6.25 μg/mL with three wells per concentration. The optical density inhibition rate was calculated using the following formula (Li et al., 2022): (the OD_600_ values of the solvent control – the OD_600_ values of the treatment) × 100%/the OD_600_ values of the solvent control. The values of 50% effective concentration (EC_50_) were calculated by regressing the optical density inhibition rate against the log of bactericide concentrations with SPSS software (version 20.0, IBM Corp., New York, USA). The experiment was repeated three times.

### 2.4. *In vivo* antibacterial assay

The protective and curative activity of 3-CCA against melon bacterial fruit blotch was assayed using a potted experiment (Li et al., 2013). The dissolved compound in acetone was diluted to final concentrations of 50, 100, and 200 μg/mL with distilled water containing 0.1% Tween-20. Seedlings of melon (hybrid melon: cv. Yangjiaomi, purchased from China Vegetable Seed Technology Co., LTD.) with two true leaves were planted in a greenhouse (25–28°C) for 10–15 days and were randomly assigned to two groups.

For the purpose of conducting a curative activity test, the cotyledons and leaves of melons in the first group were inoculated with the bacterial suspension of *Ac* (OD_600_ = 1). After inoculation for 24 h, cotyledons and leaves were sprayed with tested agents (200, 100, and 50 μg/mL) or a standard bactericide (20% thiodiazole copper SC at 100 μg/mL). For preventive activity assay, the inoculation was conducted 24 h after performing the same spraying procedure. Control seedlings within each group were inoculated with the same bacteria suspension and treated with sterile distilled water containing the same dose of acetone and Tween-20. The pots were arranged in a randomized block design with three replicates, and six melon seedlings with cotyledons injured with a sterile syringe were used per replicate.

For the development of melon bacterial fruit blotch, the inoculated melon seedlings were incubated for 7 days under the standard conditions (16 h light at 25 ± 2°C and 60 ± 5% RH + 8 h dark at 20 ± 2°C and 75 ± 5% RH), and then disease indexes were evaluated at 7 days of post-inoculation according to the coverage of the necrotic area that developed in leaves using a scale as described in Tian et al. (2015) with some modifications: namely Grade 0, healthy, asymptomatic leaf; Grade 1, the disease area accounts for approximately 5% of the leaf area; Grade 3, the disease area accounts for 6–25% of the leaf area; Grade 5, the disease area accounts for 26–50% of the leaf area; Grade 7, the disease area accounts for 51–75% of leaf area; and Grade 9, the disease area accounts for 76–100% of leaf area.


$$\begin{array}{l} \text{Disease index}=\frac{\Sigma \;\left(The\;number\;of\;diseased\;leaves\;in\;each\;grade\times \;corresponding\;grade\;value\right)}{total\;number\;of\;leaves\;investigated\times \;the\;highest\;disease\;grade\;value}\times 100\\ \text{Control effect}\left(\%\right)=\frac{Disease\;index\;in\;the\;control-Disease\;index\;in\;the\;treated\;group}{Disease\;index\;in\;the\;control}\times 100 \end{array}$$

### 2.5. Growth curve

The effect of 3-CCA on the growth of *Ac in vitro* was determined using the method described by Silva-Angulo et al. (2015) with minor modifications. The bacteria were cultured in LB broth at 180 rpm at 28°C for 18 h, then collected and inoculated into LB medium containing 3-CCA to obtain the final concentrations of 200, 100, 50, 25, 12.5, and 6.25 μg/L. Acetone (1%) was used as a solvent control. The bacteria in 96-well plates were incubated at 28°C with shaking at 180 rpm for 60 h, followed by the measurement of the optical density at 600 nm (OD_600_) every 12 h. Three independent growth assays were conducted for each concentration.

### 2.6. Bacterial morphology

Scanning electron microscopy (SEM) was performed to evaluate the effect of 3-CCA on the morphologic changes in *Ac* (Wang et al., 2016). Cultures (OD_600_ = 0.6) were centrifuged at 5,000 rpm for 5 min at 4°C and then washed three times with 0.1 mol/L of phosphate buffer (pH 7.2). The agents were added to the bacterial suspension to obtain a final concentration of 50 μg/mL. An equal volume of solvent was considered control. Then, the bacterial suspension was incubated at 28°C with shaking at 180 rpm for 24 h. The cells were collected after centrifugation at 4°C at 5,000 rpm for 5 min and then washed three times with 0.1 mol/L of phosphate buffer (pH 7.2). Samples were fixed in a 2.5% glutaraldehyde solution for 4 h at 4°C and washed with phosphate buffer three times (5 min for each time). The cells were dehydrated by a sequential grade of ethanol (30%, 50%, 70%, 80%, 90%, and 100%) for 15 min for each concentration, followed by a dehydration of 100% ethanol for 20 min. After freeze-drying for 8 h and coating with gold, the morphology of the samples was observed using an FEI Phenom desktop scanning electron microscope (FEI Company, Eindhoven, Netherlands).

Transmission electron microscopy (TEM) was used to examine the flagella formation of *Ac* according to the method described by Liu et al. (2021) with minor modifications. Ten milliliters of bacterial suspensions (OD_600_ = 0.1) were added to an LA medium containing 3-CCA (0 or 50 μg/mL) and then incubated at 28°C for 24 h. Thin sections, including the cells, were placed on a copper grid coated with carbon film, stained with 1% phosphotungstic acid two times (15 s for each time), rinsed with sterile water, and then stood for 1 h. After completely drying, the samples were observed under an HT7700 transmission electron microscope (Hitachi Ltd., Tokyo, Japan).

### 2.7. Swimming assay

Swimming assay of *Ac* was implemented according to the method of Di Bonaventura et al. (2008) with slight modifications. Cultures of *Ac* (OD_600_ = 0.6) were prepared. The LB with 0.3% agar powder was completely dissolved via heating in a microwave oven. After the temperature decreased to 40°C, 3-CCA was added to the culture medium at the final concentrations of 200, 100, 50, 25, and 12.5 μg/mL. An equal volume of acetone was used as a solvent control. The agent-contained medium was poured into sterile Petri plates. After solidification, 5 μL of bacterial suspension was drop-inoculated at the center of semisolid medium plates and incubated at 28°C for 48 h. Swimming motility was evaluated by measuring the diameter of the longest bacterial circles. The motility assays were performed three times.

### 2.8. Cell membrane permeability

The cell membrane permeability was determined by the detection of electrolyte leakage in *Ac* according to the method of Diao et al. (2014) with minor modifications. Cultures of *Ac* (20 mL) in the logarithmic growth phase were centrifuged for 20 min at 2,000 rpm. After removing the supernatant, the bacterial cells were rinsed and resuspended in 20 mL of sterile water. The 3-CCA dissolved in acetone was diluted to concentrations of 200, 100, 50, 25, 12.5, and 6.25 μg/mL with bacterial suspension. Acetone (1%) was used as a solvent control. Thereafter, the electric conductivities of these mixtures at 0, 2, 4, 6, and 8 h were measured and recorded (Kenyon et al., 1985). The experiment was repeated three times, with three replicates for each concentration.

### 2.9. EPS content

EPS content was determined according to the method of Shi et al. (2015). The acetone solution containing agents was diluted with bacterial suspension (OD_600_ = 0.6) to final concentrations of 100, 50, 25, 12.5, and 6.25 μg/mL and then incubated at 28 °C with shaking at 180 rpm for 72 h. An equal volume of acetone was used as a solvent control. The supernatant was collected after centrifugation at 3,000 rpm for 20 min at 4°C and then mixed with three volumes of absolute ethanol and incubated overnight to precipitate EPS. After centrifugation, the pellets were oven-dried at 70°C to a constant weight. Assays were repeated three times, and each assay was conducted in triplicate.

### 2.10. Biofilm formation assay

The effect of 3-CCA on biofilm formation was assessed according to a previous description (Du et al., 2018) with some modifications. The *Ac* was cultured in LB broth for 18 h at 180 rpm and 28°C. The cultures were then centrifuged and adjusted to an OD_600_ = 1. The gradient dilutions of 3-CCA were added into sterilized borosilicate glass tubes at concentrations of 100, 50, 25, 12.5, and 6.25 μg/mL (diluted with culture medium). Bacterial cultures and agent dilution buffer (100 μL of each) were added and considered negative controls. All the tubes were then incubated statically at 28°C for 7 days. Then, remove the contents from each tube gently and wash the glass tubes three times with sterile deionized water. After that, the biofilms contained in the glass tubes were stained with 0.1% crystal violet solution for 1 h, rinsed three times with deionized water to diminish the unbound crystal violet, and then stained with 10% glacial acetic acid for 1 h. The solution of 200 μL from each tube was transferred to 96-well plates to measure the OD_600_ values. The inhibition rate (%) was calculated as: (the OD_600_ values of the control- the OD_600_ values of the treatment) × 100/the OD_600_ values of the control. Six test tubes were prepared for each concentration treatment, and the experiment was repeated three times.

### 2.11. Statistical analysis

Results were expressed as means ± standard error (SE) of three independent repeated experiments. Data were analyzed with a variance analysis using SPSS software (version 20.0, IBM Corp., Armonk, NY, USA). Duncan's multiple range test was conducted to compare statistical significance at a *P* < 0.05. Graphs were generated with Sigma Plot (version 12.5, Systat Software Inc., San Jose, CA, USA).

## 3. Results

### 3.1. Antibacterial screening of 3-CCA

**Figure 1:**
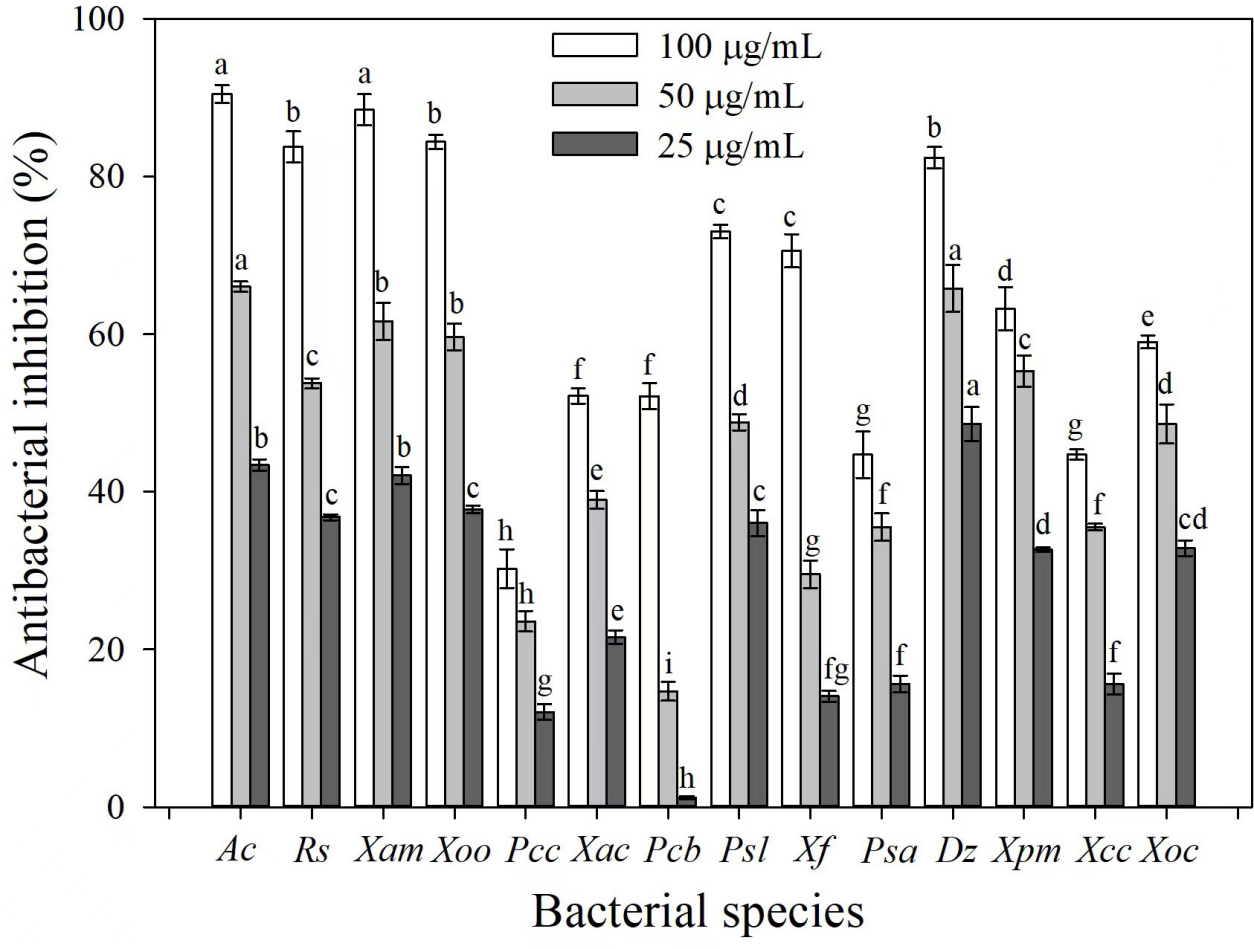
Antibacterial activities of 3-CCA against different bacterial species. Values presented are means ± standard errors from three independent experiments. Different letters on top of the bars within each dosage group indicate significant differences in the inhibition rate against different bacterial species according to Duncan's multiple range test at a *P* < 0.05.

The antibacterial activities of 3-CCA against 14 phytopathogenic bacterial species were determined. Results showed that 3-CCA had antibacterial activities against almost all the tested strains except *Pcc* (Figure 1). Among them, *Ac* was the most sensitive species to 3-CCA, showing an inhibition of 90.45% at a concentration of 100 μg/mL (Figure 1). In addition, inhibition rates of 3-CCA at 100 μg/mL against *Xam, Rs, Xoo*, and *Dz* were 88.45%, 84.39%, 83.76%, and 82.38%, respectively (Figure 1).

Based on the antibacterial screening results, the EC_50_ values of 3-CCA against these five sensitive bacteria were evaluated. The results demonstrated that the EC_50_ values of 3-CCA against *Ac, Xam, Rs, Xoo*, and *Dz* were 26.64, 33.17, 31.62, 35.98, and 40.73 μg/mL, respectively (Table 1).

**Table 1:** Toxicity of 3-CCA against five different bacterial strains.

**Bacteria**	**Lc-P**	**r**	**EC_50_ (μg/mL)**	**95% confidence limits**
*Ac*	y = 3.0281 + 1.3833x	0.9647	26.64	18.69–37.97
*Rs*	y = 2.3754 + 1.7497	0.9656	31.62	23.86–41.91
*Xam*	y = 2.1389 + 1.8813x	0.9838	33.17	25.40–43.33
*Xoo*	y = 2.2518 + 1.7661x	0.9767	35.98	27.21–47.57
*Dz*	y = 2.2011 + 1.7385x	0.9661	40.73	30.74–53.96

Lc-P, logarithmic concentration-probit line; r, correlation coefficient.

### 3.2. Efficacy of 3-CCA for controlling *Ac*

**Figure 2:**
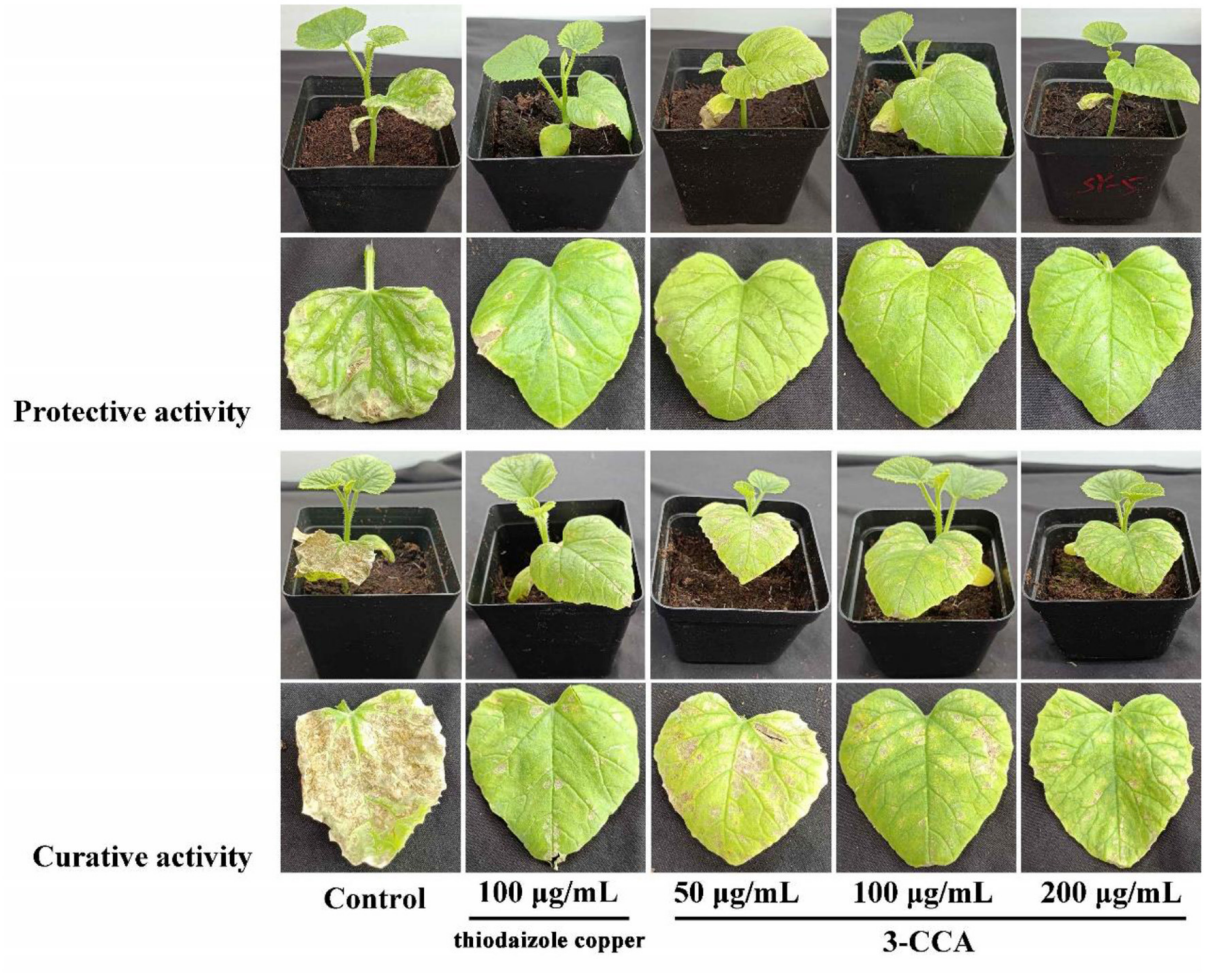
*In vivo* protective and curative effects of 3-CCA against bacterial fruit blotch caused by *Ac* using potted melon plants in the greenhouse. The experiments were performed three times.

The present results showed that 3-CCA had both protective and curative effects against *Ac* (Figure 2). The protective effect was superior to the curative effect (Figure 2). The protective and curative effects of 3-CCA at 200 μg/mL against *Ac* were 61.88% and 53.19% (Table 2), which were better than those of the positive control thiodiazole copper.

**Table 2:** *In vivo* protective and curative effects of 3-CCA against *Ac*.

**Sample**	**Concentration (μg/mL)**	**Protective effect**		**Curative effect**	
		**Disease index**	**Control effect (%)**	**Disease index**	**Control effect (%)**
3-CCA	200	21.6 ± 2.14d	61.88a	27.16 ± 2.14c	53.19a
	100	28.35 ± 0.98bc	50.02bc	32.1 ± 1.07b	44.66b
	50	30.25 ± 2.14b	46.74c	35.19 ± 3.7b	39.17c
Thiodiazole copper	100	27.4 ± 1.32c	51.76b	32.1 ± 2.14b	44.73b
Control	/	56.79 ± 2.14a	/	58.03 ± 2.14a	/

Means in a column followed by different letter are significantly different (*P* < 0.05) according to Duncan's multiple range test.

### 3.3. Effect of 3-CCA concentration on the growth of *Ac*

**Figure 3:**
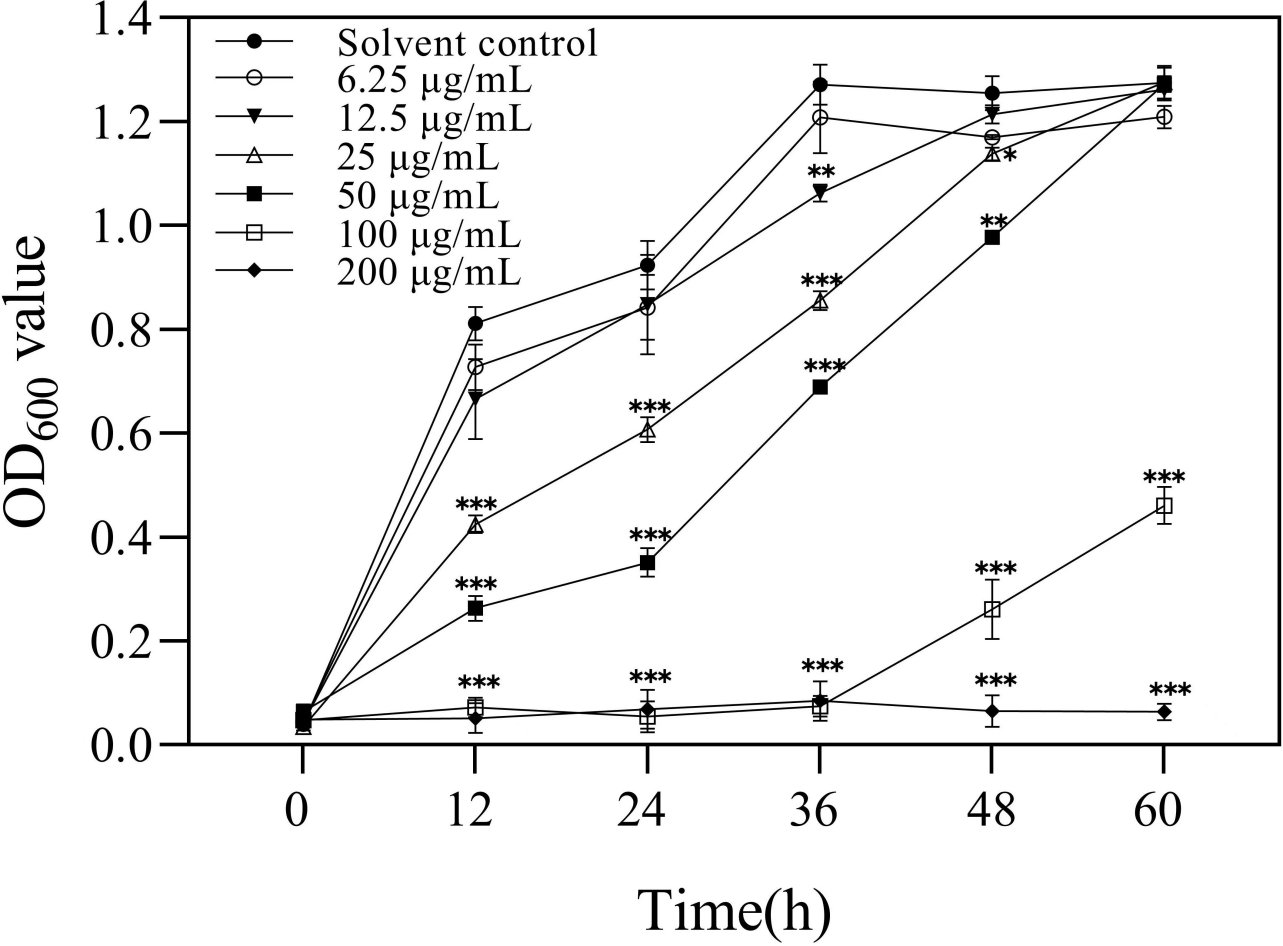
Bacterial growth curve of *Ac* in the presence of 3-CCA at concentrations ranging from 6.25 to 200 μg/mL. The solvent was indicated as a negative control. Values presented are means ± standard errors from three independent experiments. *P < 0.05, **P < 0.01, and ***P < 0.001 (Bonferroni's multiple comparisons test).

A growth curve assay was performed to analyze the effect of 3-CCA concentrations on the growth of *Ac*. As shown in Figure 3, 3-CCA at concentrations ranging from 25 to 200 μg/mL caused a decrease in the growth rate when compared to the solvent control, which was obviously dependent on concentrations. The mean values of the lag phase at 60 h are significantly inhibited by 3-CCA at concentrations of 100 and 200 μg/mL.

### 3.4. The effect of 3-CCA on the cell morphology of *Ac*

**Figure 4:**
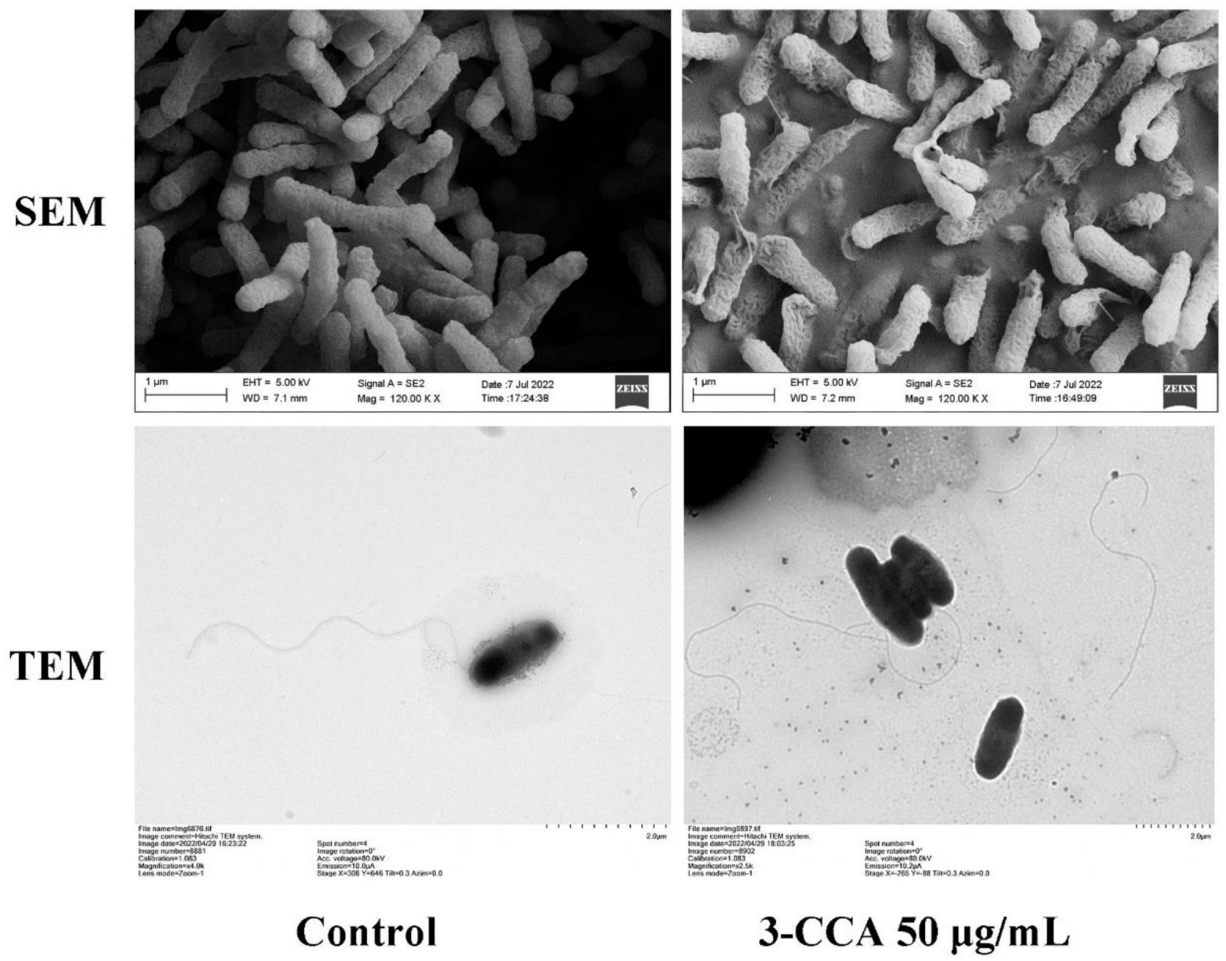
SEM and TEM observations of the *Ac* treated with 3-CCA.

The morphological changes in *Ac* after treatment with 3-CCA were investigated via SEM and TEM. As shown in Figure 4, the control bacterial cells maintained their uniformity in shape, showing a plump and smooth form. Comparatively, the surfaces of the bacteria were rough and wrinkled after they were treated with 50 μg/mL of 3-CCA (Figure 4). TEM results displayed that the polar flagella of *Ac* in solvent control grew normally, but some of the flagella were broken after being exposed to 50 μg/mL 3-CCA (Figure 4).

### 3.5. Effect of 3-CCA on the bacterial motility of *Ac*

**Figure 5:**
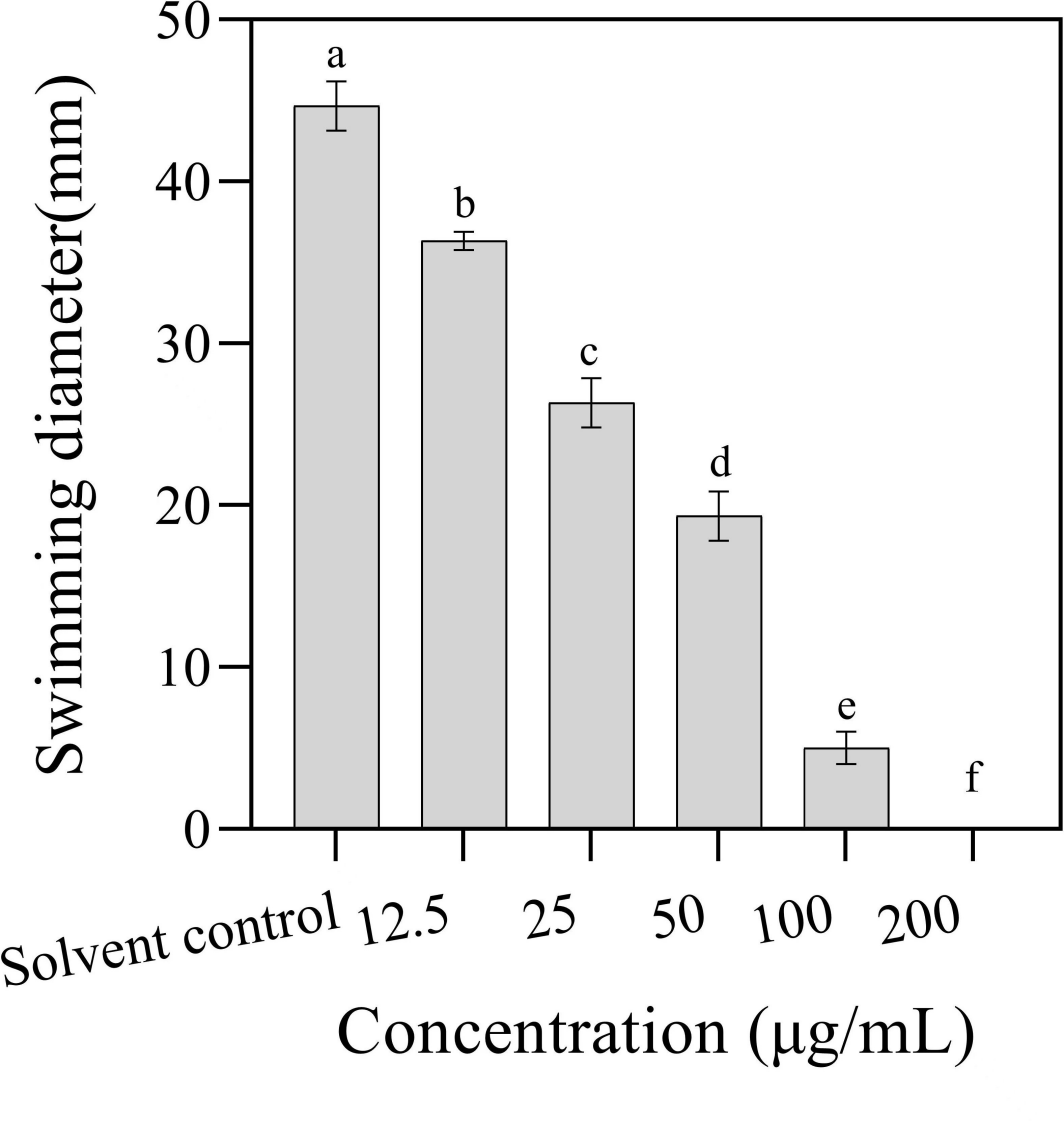
Effects of 3-CCA at different concentrations on the swimming motility of *Ac*. Values presented are means ± standard errors from three independent experiments. Different letters on top of the bars indicate that the means are significantly different according to Duncan's multiple range test at a *P* < 0.05.

The effect of 3-CCA on the bacterial motility of *Ac* was determined by plate inhibition assay. As shown in Figure 5, the swimming diameters of the colonies decreased along the increases in agent concentrations, in which the swimming diameters of *Ac* exposed at 12.5 to 100 μg/mL of 3-CCA being 18.79% to 88.81% lower than those in control. No swimming motility was observed when the concentration of 3-CCA increased to 200 μg/mL.

### 3.6. The effect of 3-CCA on the cell membrane permeability of *Ac*

**Figure 6:**
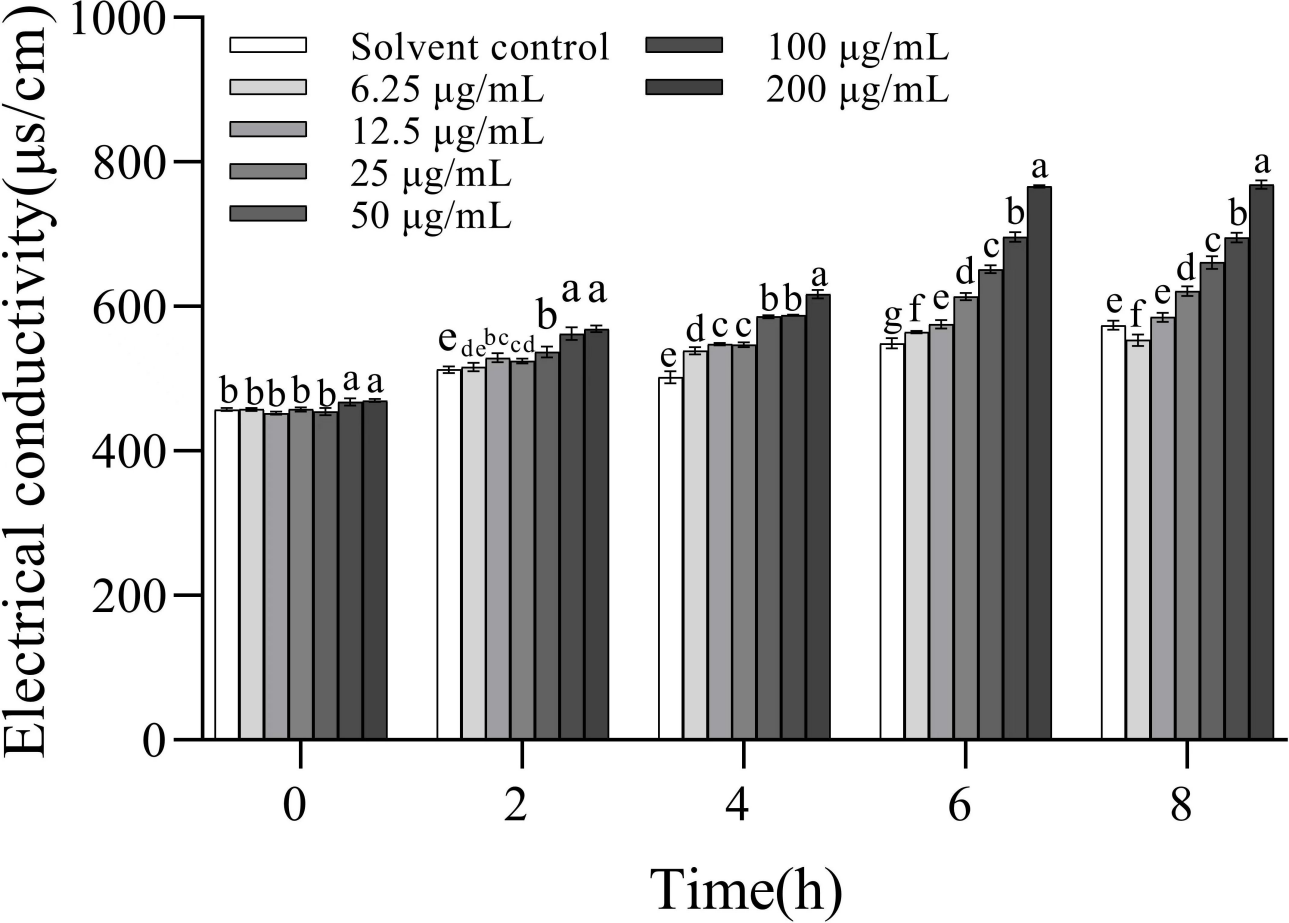
Changes in membrane permeability of *Ac* after treatment with 3-CCA. Values presented are means ± standard errors from three independent experiments. Different letters on top of the bars within each time group indicate significant differences in the electric conductivity of *Ac* treated by different dosages according to Duncan's multiple range test at a *P* < 0.05.

The electric conductivity values increased with increasing concentrations and the extension of incubation time, indicating that 3-CCA promoted the increase in membrane permeability of *Ac* in a dose- and time-dependent manner (Figure 6). When the treatment concentrations ranged from 25 to 200 μg/mL, the electric conductivities were significantly higher than those of the solvent control group. 3-CCA at 6.25 or 12.5 μg/mL exerted a minimal effect on membrane permeability compared to other concentrations.

### 3.7. The effect of 3-CCA on the EPS content of *Ac*

**Figure 7:**
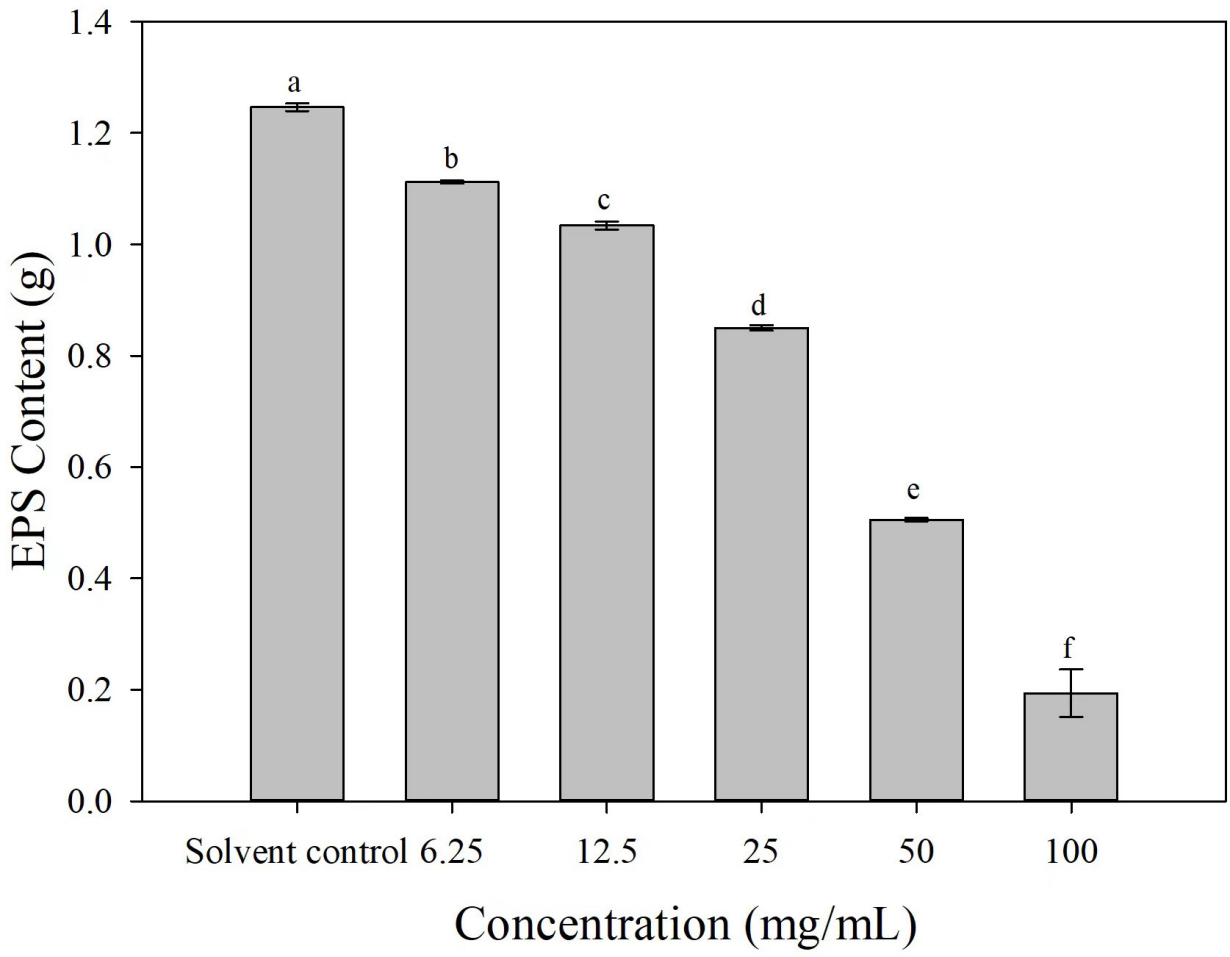
Effects of 3-CCA at different concentrations on the EPS production of *Ac*. Data represent means ± SE of three independent experiments. Different letters on top of the bars indicate that the means are significantly different according to Duncan's multiple range test at a *P* < 0.05.

The EPS production was decreased to varying degrees after the cells of *Ac* were treated with 3-CCA at different concentrations. Compared with solvent control, the production of EPS of *Ac* treated with 3-CCA at all concentrations was significantly reduced (*P* < 0.05) (Figure 7).

### 3.8. The effect of 3-CCA on the biofilm formation of *Ac*

**Figure 8:**
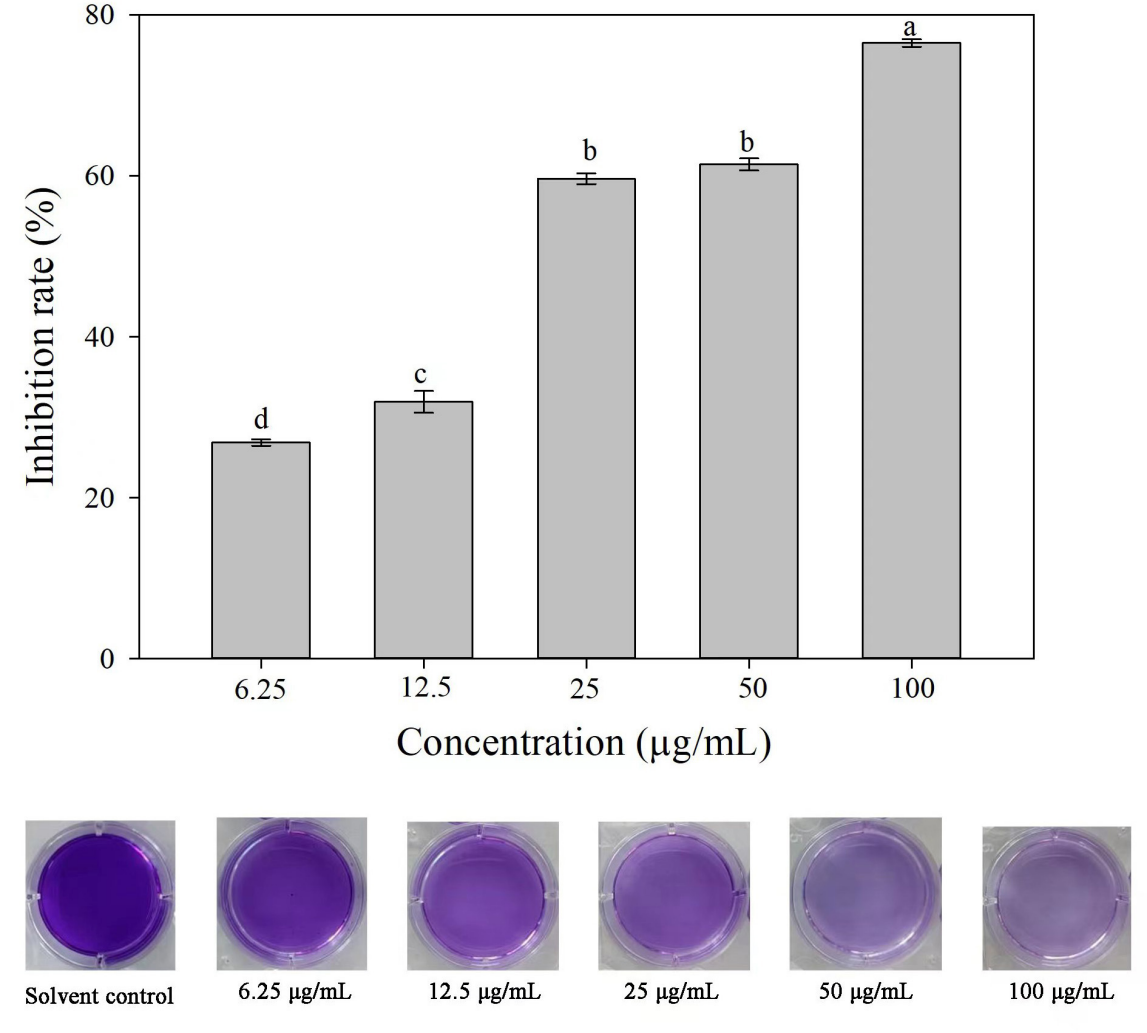
Effects of 3-CCA at different concentrations on the biofilm formation of *Ac*. Data represent means ± SE of three independent experiments. Different letters on top of the bars indicate that the means are significantly different according to Duncan's multiple range test at a *P* < 0.05.

Compared with the solvent control, 3-CCA resulted in remarkable inhibition of biofilm formation, and the inhibitory effect was promoted along with increased concentrations of 3-CCA (Figure 8). The average inhibition rates of the 3-CCA treatment at 100 and 50 μg/mL were 76.48% and 62.13%, respectively (Figure 8).

## 4. Discussion

The occurrence of diseases caused by plant-pathogenic bacteria has been one of the most important problems in plant protection (Mansfield et al., 2012). With the enhancement of consumers' awareness of environmental protection and increasing demand for safe agricultural products, the development of new chemicals with high efficiency and environmental friendliness is imminent. 3-CCA is a coumarin compound and possesses diverse pharmacological activities, including anticoagulant, antioxidant, antibacterial, and antifungal properties (Barot et al., 2015). In the present study, the *in vitro* antibacterial effect of 3-CCA against 14 pathogenic bacteria was evaluated. The results showed that 3-CCA had broad-spectrum antimicrobial activity against almost all tested bacteria, with inhibition rates ranging from 31.03% to 93.11%. According to the result of the toxicity assay, the EC_50_ values of 3-CCA against five tested bacteria ranged between 26 and 41 μg/mL, which was in accordance with the report of Chen et al. (2016), who demonstrated the antibacterial effect of coumarins against *Rs*. The results of pot experiments showed excellent protective and curative effects of 3-CCA on melon bacterial fruit blotch disease. These findings suggest that 3-CCA could be a potential bactericide to control plant disease.

Although the compound 3-CCA has shown inhibitory activity against most of the pathogens used in our study, its activity against *Ac* was significantly higher than other bacterial species (Figure 1). Thus, the present study illustrates the mode of action of 3-CCA against *Ac*. Generally, the mode of mechanism of some bactericides involves the disruption of membrane integrity (Feng et al., 2021). Many natural bactericidal components, such as Biochanin A (Hu et al., 2021), daphnetin (Yang et al., 2016), and methyl gallate (Fan et al., 2013), have been tested to inhibit the growth of bacteria by destroying the cell membranes. In this study, we found that the bacteria treated with 3-CCA had a seriously wrinkled surface along with increasing conductivity in a dose-dependent way. In addition, 3-CCA was found to disrupt flagellar growth and block bacterial motility. As the main organelle of bacterial motility, the bacterial flagellum is a quite complex nanomachine that contributes to both bacterial adherence and biofilm formation (Khider et al., 2018; Zhou et al., 2020). Flagellar motility has been demonstrated to play an important role in the initial stage of bacterial biofilm formation (Houry et al., 2010). Without the flagella, *Agrobacterium tumefaciens* cannot form biofilms (Sato et al., 2005; Waege et al., 2010; Ding et al., 2017). EPS are major components of bacterial biofilm and play a crucial role in the formation and adhesion of bacterial biofilm (Gacesa, 1998). The present study demonstrated that 3-CCA inhibited the biofilm formation of *Ac* and reduced the production of EPS, thus contributing to the inhibited formation of biofilms and reduced adhesion in bacteria. We deduced that 3-CCA might exert bactericidal effects against *Ac* by disrupting the membrane integrity while blocking biofilm formation by interfering with flagellum formation and decreasing EPS production. As 3-CCA also exhibited inhibitory activity against other bacterial pathogens, it needs to be experimentally validated if there is a similar mode of action on the other pathogens in our future study.

Moreover, we found that 3-CCA could inhibit the biofilm formation of *Ac* at lower concentrations (6.25–25 μg/mL). In phytopathogenic bacteria, biofilm and EPS act as essential pathogenicity factors that are closely related to the pathogenic forces (Lim et al., 2017). The formation of bacterial biofilms can enhance the resistance of bacteria to bactericides, decrease their drug sensitivity, and improve their adaptability and tolerance to the external environment, which increases the difficulty of controlling bacterial diseases (Vestby et al., 2020). Inhibiting the formation of pathogen biofilm has recently been considered a novel strategy for disease control (Harding et al., 2019). Prithiviraj et al. (2005) found that salicylic acid could inhibit biofilm formation in the roots of *Arabidopsis thaliana*. Harding et al. (2019) discovered that treatment with oxysilver nitrate effectively reduced bacterial biofilm formation, indicating that this compound could be used as a candidate for controlling seed-transmitted bacterial diseases. In this study, 3-CCA remarkably inhibited the biofilm formation of *Ac* in a concentration-dependent manner, which was consistent with the report of Yang et al. (2016), who confirmed an inhibition of hydroxycoumarins on the biofilm formation of *Rs*. In addition, one of the roles of coumarin compounds could be to block bacterial quorum sensing (Reen et al., 2018). Hence, we proposed that the inhibition of biofilm formation by bacteria by 3-CCA could be associated with the block of quorum sensing, which needs further study for validation. The findings suggested that 3-CCA might be a promising lead compound for further structure optimization to develop novel antibacterial agents for controlling plant diseases. However, some researchers reported that excess consumption of coumarins may adversely affect our health because they are easily absorbed from the intestines into the lymph and blood, causing cirrhosis of the liver (Kowalczyk et al., 2020), so the toxicity of 3-CCA or novel chemicals designed based on this lead compound to other non-target organisms should also be carried out in future.

In summary, 3-CCA showed a broad-spectrum and efficient bactericidal effect against plant bacterial pathogens, including *Ac*. 3-CCA exerted an inhibitory effect against *Ac* by disrupting the integrity of cell membranes and preventing biofilm formation. The results suggest that 3-CCA may be a potential natural lead compound. The antibacterial molecular mechanisms of 3-CCA and its structural optimization need to be further investigated to explore novel antibacterial agents.

## Data availability statement

The original contributions presented in the study are included in the article/supplementary material, further inquiries can be directed to the corresponding authors.

## Author contributions

GF, JZ, and F-DZ designed the experiments, analyzed the data, and wrote the manuscript. XF, L-SG, and H-CY performed the experiments. H-XD and Y-XG contributed to reagents and analysis tools. All authors contributed to the article and approved the submitted version.
